# The Early Identity Exploration Scale—a measure of initial exploration in breadth during early adolescence

**DOI:** 10.3389/fpsyg.2015.00533

**Published:** 2015-04-30

**Authors:** Maria Kłym, Jan Cieciuch

**Affiliations:** Institute of Psychology, Cardinal Stefan Wyszyñski University in WarsawWarsaw, Poland

**Keywords:** identity exploration, early adolescence, early identity exploration scale (EIES), identity formation

## Abstract

The existing models and measurement instruments concerning identity appear to primarily focus on adolescence and early adulthood, and studies extending identity research to younger stages of life are scarce. There has been a particular lack of instruments measuring the early stages of identity formation, especially the process of exploration, which has been portrayed as a central process during this particular period of life. Our aim is to help fill the gap in the literature and facilitate further studies of the exploration process by providing an appropriate instrument to measure exploration in breadth during early adolescence. As a coherent and mature sense of identity is closely associated with psychosocial well-being, an effective identity exploration scale will enable researchers to assess the predictors of young adolescents' well-being. We propose a model of identity exploration domains based on the literature and considering 12 exploration domains: physical appearance, free time, family, work, boyfriend-girlfriend relationships, own opinion formation, perception of own place in the life cycle, self-reflection, future, future family, outlook on life, and attitude toward rules. The study was conducted on a group of *N* = 454 adolescents (50% males, *M*_age_ = 13.04, *SD* = 0.98). Both reliability and structural validity, as verified by confirmatory factor analysis were satisfactory. The instrument is invariant across gender groups at the scalar level of measurement invariance.

## Introduction

Identity formation is an important lifelong process—it begins in childhood, becomes particularly important during adolescence, and continues throughout life (Erikson, 1950; Luyckx et al., 2005; Schwartz et al., 2011). Erikson describes identity as a response to the question “Who am I?” In other words, identity denotes an integrated and cohesive sense of self that endures and continues to develop as we age. In the Eriksonian tradition, identity is also defined as a self-theory (Berzonsky, 2011).

Current models of identity are based on the work of Erikson's follower Marcia (1966), who describes identity formation as consisting of two qualitatively distinct processes: exploration and commitment. Exploration (initially called *crisis)* is defined as an “adolescent's period of engagement in choosing among meaningful alternatives,” and commitment refers to “the degree of personal investment the individual exhibits” (Marcia, 1966, p. 551). Over the past several decades, numerous studies demonstrate the existence of links between the identity dimensions and a number of personality and social variables, including well-being (Marcia, 1993; Luyckx et al., 2005; Crocetti et al., 2008a; Karaś et al., 2013). Well-being can be treated as an effect of mature identity achievement, built through processes of exploration and commitment. Thus, measurement of these processes early in identity development may have important implications for understanding the early predictors of well-being.

Both processes can be assessed by well-established instruments within various current models of identity formation. A serious limitation of such measurement instruments, however, is that they are intended for studies concerning adolescence or perhaps early adulthood, so the periods when both processes occur and are already fairly well established. What is lacking is an instrument that could be used to measure emerging exploration during the period between childhood and adolescence. Such an instrument should take into account both the developmental specificity of children entering adolescence and the specificity of freshly forming exploration. The article aims to develop such an instrument that includes those requirements.

### Identity determinants of well-being

The theoretical writings of Erikson (1959) and modern empirical studies recognize the important role of identity formation in achieving well-being. It is reported that commitment and exploration are both significant in terms of their effects on well-being. As the literature indicates (Crocetti et al., 2008a,b), individuals with a high level of identity commitment were characterized by a low level of psychosocial problems and less anxiety and depression in their reactions (Luyckx et al., 2007). They are also more heavily socially adjusted (Luyckx et al., 2006). Recent research also confirms that the dimensions of identity, including exploration, constitute strong predictors of various well-being dimensions (inter alia: Waterman et al., 2010; Karaś et al., 2013; Kłym et al., 2014). Berzonsky and Cieciuch (2014) demonstrate that eudaimonic well-being is determined by the identity styles, which can be interpreted as styles of identity exploration. Although the effect was mediated by commitment, the contribution of informative exploration remained significant on nearly all dimensions of well-being in Ryff's (1989) conceptualization (Berzonsky and Cieciuch, 2014). However, as well-being is located in Marcia's achieved identity status, accomplishing well-being as an adult involves performing both processes: exploration and then commitment. Thus, investigation of the exploration, which is the initial phase of identity formation is looking for early predictors of well-being.

### Existing identity exploration instruments

The most commonly used instruments for measuring identity formation including exploration process are the Utrecht-Management of Identity Commitments Scale (U-MICS; Crocetti et al., 2008a), Dimensions of Identity Development Scale (DIDS; Luyckx et al., 2006, 2008), and Identity Style Inventory (ISI; Berzonsky, 1990). The U-MICS measures three identity dimensions (in-depth exploration, commitment, and reconsideration of commitment). In-depth exploration is defined as adolescents' reflections on their choices and the active consideration and management of current commitments (Crocetti et al., 2008b). The DIDS is based on a five-dimensional identity model containing the following constructs: exploration in breadth, exploration in depth, commitment making, identification with commitment, and ruminative exploration. Thus, in the DIDS, the exploration dimension is subdivided to reflect the positive and negative aspects of functioning. The authors have distinguished dysfunctional and maladaptive ruminative exploration from two other exploration dimensions: exploration in breadth, which is understood as discovering, investigating, and gathering information on the various identity alternatives that lead to commitment; and exploration in depth, which as in Crocetti et al. (2008a,b) definition, is understood as potentially collecting the most complete information possible on the current decisions and commitments that an individual has already made.

The ISI was developed by Berzonsky (1990) to measure three identity styles defined as social-cognitive strategies, which individuals employ in the construction of their identities and serve to differentiate among individual identities. They include the informational style, the normative style, and the diffuse-avoidant style, and these could be regarded as three forms of exploration (Berman et al., 2001).

The existing models of identity formation and related measurement instruments appear to focus on identity formation in adolescence and early adulthood. Some are extended to describe adults (e.g., Whitbourne, 1986). However, studies extending the literature on identity to younger stages of life are scarce. There has been a particular lack of instruments measuring the early stages of identity formation, especially the process of exploration, which is the first step in identity formation. The youngest ages at which existing scales have been applied can be found in studies employing the U-MICS. Research presented by Crocetti et al. (2008a,b) was conducted on a group of early adolescents aged 10–13 years (*M*_age_ = 12.4; *SD* = 0.5), and Klimstra et al. (2010) conducted their research on two groups of adolescents aged 12–20 (the descriptive statistics for younger participants are *M*_age_ = 12.4; *SD* = 0.59). However, the identity processes measured by the U-MICS concern exploration which occurs after the first commitment—it is an in-depth exploration. Hence, the model does not fully capture the process of exploration in breadth. Exploration in breadth is included in the DIDS, but it concerns general plans for the future, and no study to date applies the DIDS to early adolescents. Furthermore, Berzonsky's ISI questionnaire measuring identity styles is typically applied to groups of participants no younger than 16–17 years of age (Berzonsky et al., 2013), with only one study considering early adolescents aged over 12 (Berzonsky et al., 2007).

In summary, existing measures were developed for adolescents, and hence, their use with younger participants remains experimental. The main problem is that the existing instruments are not suitable for studies on people in the period of late childhood and early adolescence. There are at least two reasons why these instruments are not suitable for such studies. The first concerns the form of the items used in the questionnaires, and the second concerns the use of a process approach rather than a domain approach. Regarding the first problem, the items in the existing identity formation instruments require participants to have skills in thinking abstractly and generalizing various thoughts and behaviors. Regarding the second problem, the conceptualization of the exploration process in these instruments in some way assumes that the process is similar across various domains of life. This assumption seems to be much less likely to hold in the period when the process of exploration is only beginning (that is, at the threshold of early adolescence).

Our aim is to help fill the gap in the literature and facilitate further studies of the exploration process by providing an appropriate instrument for measuring exploration in breadth during early adolescence. The proposed instrument addresses the limitations mentioned above: it does not require abstract thinking abilities, and it measures exploration in different life domains.

### Defining domains of exploration

In Marcia's research tradition, exploration is the process of considering and choosing among meaningful alternatives (Marcia, 1966) as well as the active questioning and weighing of various identity options. Moreover, there is variation in the degree to which adolescents search for different alternatives with respect to their goals, values, and convictions (Luyckx et al., 2008). This process involves exploring and discovering who and what a person can be and is followed by commitment making and engagement.

The initiation of identity formation in early adolescence, as manifested in exploration in breadth, is closely related to growing autonomy (Weeks and Pasupathi, 2010), the consideration of one's future as an independent entity and establishing one's first romantic relationships (Furman and Shaffer, 1999). All of the above are developed on the basis of the sense of competence that is gained in earlier developmental stages (Erikson, 1950) and leads to increasing self-strength in individuals.

As there were no previous attempts to investigate particular exploration areas in the literature, we proposed to conceptualize and measure exploration in early adolescence. Based upon theoretical consideration and previous empirical studies, we proposed to distinguish 12 domains in which exploration in breadth can occur in early adolescence. These domains are presented in Table 1.

**Table 1:** **Domains of exploration**.

**Domain of exploration**	**Description**
Physical appearance	Beginning to draw attention to appearance, seeking own style; the extent to which the physical self becomes a persistent presence (Brinthaupt and Lipka, 2002).
Free time	Activities that the early adolescent engages in or would like to engage in during free time to find his or her interests and passions as well as to discover strengths, as expressed in their own actions (Erikson, 1968).
Family	Reflections on the family of origin, the prevailing relations and the relevance or similarity to family members; comparing one's own family with peers' families (McKinney and Renk, 2011).
Work	Considerations regarding what the early adolescent wants to do in adult life, including ideas about what profession would be the most suitable for him or her (Marcia, 1966).
Boyfriend-girlfriend relationships	Drawing attention to the opposite sex, interest in romantic relationships, thinking about which partner would be best suited to him or her and what type of relationship one would like to create (Furman and Shaffer, 1999).
Perception of own place in the life cycle	The early adolescent's impression that he or she is no longer a child and feelings of discomfort in situations in which others (especially parents) treat him/her as a child. A sense of “growing out” of childhood, entering a new phase, and “fully living in these ‘new clothes”’ (Brinthaupt and Lipka, 2002).
Self-reflection	Thinking about him/herself and asking questions about who he or she is. The desire to discover new things about him/herself and attempts to become further acquainted with him/herself (Brinthaupt and Lipka, 2002).
Future	Consideration of the various directions that one could take in life. Pondering how he or she would like to live, which life goals are important and what type of lifestyle would be appropriate for him or her in the future (Luyckx et al., 2006, 2008).
Future family	Imagining and thinking about the family that one will create in the future, the relationships between the members of the family and the manner in which he/she would like the family to function (Furman and Shaffer, 1999).
Outlook on life	Considering different value systems, searching for information and reflections to justify and intensify his/her beliefs, doubts regarding one's beliefs (Erikson, 1950; Boyes and Chandler, 1992).
Attitude toward rules	Pondering whether all rules, orders and prohibitions are necessary and make sense and considering what would happen if the early adolescent had not acquiesced to such rules (Magnusson et al., 1985; Krettenauer et al., 2013).

### Development of the early identity exploration scale

To measure exploration in the areas listed in Table 1, we developed the Early Identity Exploration Scale (EIES). In our research, we focus on early adolescence; therefore, a traditional questionnaire exclusively consisting of classic self-reported items could be excessively difficult for the young participants. In situations in which providing optimal responses to questionnaire items requires substantial cognitive effort, certain respondents might provide insufficient answers (Krosnick, 1991). Therefore, the tool should be structured to ensure its suitability for the developmental period (the tool should not be more cognitively demanding in terms of self-reflection than the questionnaires applied in adult studies).

Thus, in the EIES, we first employed portrait descriptions and then presented items that refer to these portraits. Such portraiture enables participants to compare themselves to described persons and facilitates self-reflection. A set of items for each of the scales was preceded by a brief description of two persons (according to the participant's sex) differing from one another with respect to what they do, feel and think in a particular field to simplify the participants' identification with one of them and thus to ensure that the items are fully understandable.

In each scale, after such an introduction, a participant views a set of 4–7 items and estimates the frequency of thoughts/feelings/acts described by each item on a 5-item Likert scale (from *very rarely or never* to *very often or always*). The final item selection was derived from a discussion with a group of seven developmental psychologists. The full EIES questionnaire can be found in Appendix 1 (Please note that there are two versions of the EIES—according to participant's sex: version for boys with male names in descriptions and version for girls with female names. Appendix 1 is EIES version for boys).

The main research goal of the current study was to assess the psychometric properties of the EIES. In particular, we aimed to (1) determine the reliability of each of the 12 proposed scales, (2) confirm the distinguishability of the 12 proposed scales in a factor analysis and (3) verify the measurement invariance of the scales across gender groups. Based on the literature, we distinguished 12 domains where exploration can appear. This catalog is neither final nor complete. In future studies, it would be worthwhile to remove some of the domains or to add new ones. Through the empirical analysis in this study, we verified the proposed 12-scale measurement model in confirmatory factor analysis (CFA). In addition, we conducted exploratory factor analysis (EFA) on the 12 scales to answer the question whether the 12 identified scales are grouped into certain sets of domains.

## Method

### Participants and procedure

The total group of respondents consisted of 454 adolescents (50% males) between 11 and 14 years of age (*M* = 13.04, *SD* = 0.98). The age distribution was as follows: 11 year olds represented 9% of the total group, 12 year olds 20%, 13 year olds 30%, and 14 year olds 41%.

Group studies were conducted during school lessons using a traditional pen-and-paper method. All of the participants were primary (5th and 6th grade) and secondary (1st and 2nd grade) school pupils from one large (1.7 million inhabitants) and one small town (over 30 thousand inhabitants) in central Poland. We endeavored to satisfy all ethical principles required when working with minors (Greig et al., 2013). The study was anonymous and the participation of the young adolescents required their parents' written consent. The children received complete information regarding the research and its aim (including who will have access to the data and what will be done with the data when the research is complete) to provide their informed consent to participate; that participation was freely volunteered. The children were also aware that they could withdraw at any time. Finally, the degree of confidentiality was explained to the children at the outset of the research.

### Analysis

As conceptualization has been performed and 12 domains of exploration have been distinguished, the EIES questionnaire was constructed deductively to measure the given areas. Thus, the appropriate method to test the factorial validity is CFA. Additionally, we examined the measurement invariance across girls and boys via a multigroup confirmatory factor analysis (MGCFA). We conducted all factorial analyses in the Mplus 7.1 statistical software. While running the CFA, we employed weighted least squares with an adjusted mean and variance estimator. We evaluated the model fit based on the cut-off proposed in the literature, thus as Hu and Bentler (1999) and Marsh et al. (1999) suggest, we treated RMSEA (root mean square error of approximation) < 0.08 and CFI (comparative fit index) > 0.90 as an indicator of acceptable model fit.

Three levels of measurement invariance were tested using the MGCFA. The first level, *configural invariance*, assumes the same pattern of item-factor loadings occurring across compared groups. *Metric invariance* requires, furthermore, that unstandardized factor loadings are invariant across groups. *Scalar invariance* requires all the conditions of configural and metric invariance to be established, and it assumes that the scale's item intercepts are invariant across groups. The configural level allows a general comparison of the structure across groups. The metric level allows a comparison of correlates of the variables because it supports the same meaning of the measured constructs across groups. Scalar measurement invariance is a precondition of any meaningful means comparison across groups (Davidov et al., 2014). According to Chen's (2007) recommendation, we treated a difference of <0.01 in the CFI index and a difference of <0.015 in the RMSEA between the configural and metric levels as an indicator of metric measurement invariance and between the metric and scalar levels as an indicator of scalar measurement invariance.

Missing data were scarce, with a rate ranging from 0.4 to 2.2% for the items. The only exception was item 60, with a 3.3% missing-data rate. Missing data were handled by default in Mplus.

Additionally, we used EFA to verify whether the exploration domains in the 12 scales cluster in any meaningful manner.

## Results

### Reliability

The Cronbach alpha reliability coefficients for the scales ranged between 0.62 and 0.91. All coefficients are presented diagonally in Table 2 below.

**Table 2:** **Correlations between the variables measured by the EIES (correlations between observed variables are above the diagonal, and correlations between latent variables are below the diagonal) and Cronbach's alpha on the diagonal (in bold)**.

	**(1)**	**(2)**	**(3)**	**(4)**	**(5)**	**(6)**	**(7)**	**(8)**	**(9)**	**(10)**	**(11)**	**(12)**
(1) Physical appearance	**0.73**	0.44^**^	0.45^**^	0.37^**^	0.35^**^	0.30^**^	0.15^**^	0.48^**^	0.33^**^	0.35^**^	0.33^**^	0.26^**^
(2) Free time	0.58^***^	**0.79**	0.31^**^	0.52^**^	0.18^**^	0.19^**^	0.04	0.44^**^	0.42^**^	0.34^**^	0.40^**^	0.15^**^
(3) Family	0.58^***^	0.40^***^	**0.79**	0.37^**^	0.23^**^	0.42^**^	0.17^**^	0.52^**^	0.32^**^	0.36^**^	0.36^**^	0.37^**^
(4) Work	0.47^***^	0.64^***^	0.46^***^	**0.85**	0.26^**^	0.24^**^	0.22^**^	0.50^**^	0.63^**^	0.49^**^	0.43^**^	0.27^**^
(5) Boyfriend-girlfriend relationships	0.43^***^	0.23^***^	0.26^***^	0.29^***^	**0.85**	0.28^**^	0.40^**^	0.36^**^	0.27^**^	0.44^**^	0.28^**^	0.30^**^
(6) Own opinion formation	0.37^***^	0.24^***^	0.52^***^	0.28^***^	0.32^***^	**0.83**	0.54^**^	0.43^**^	0.34^**^	0.25^**^	0.29^**^	0.52^**^
(7) Perception of own place in the life cycle	0.18^**^	0.07	0.20^***^	0.26^***^	0.46^***^	0.62^***^	**0.89**	0.27^**^	0.31^**^	0.30^**^	0.19^**^	0.35^**^
(8) Self-reflection	0.60^***^	0.54^***^	0.62^***^	0.59^***^	0.40^***^	0.49^***^	0.31^***^	**0.91**	0.62^**^	0.55^**^	0.58^**^	0.41^**^
(9) Future	0.41^***^	0.51^***^	0.38^***^	0.72^***^	0.32^***^	0.40^***^	0.35^***^	0.69^***^	**0.90**	0.48^**^	0.50^**^	0.30^**^
(10) Future family	0.44^***^	0.41^***^	0.42^***^	0.57^***^	0.48^***^	0.29^***^	0.35^***^	0.61^***^	0.55^***^	**0.85**	0.48^**^	0.36^**^
(11) Outlook on life	0.45^***^	0.53^***^	0.47^***^	0.55^***^	0.33^***^	0.37^***^	0.23^***^	0.72^***^	0.63^***^	0.56^***^	**0.78**	0.37^**^
(12) Attitude toward rules	0.37^***^	0.24^***^	0.54^***^	0.35^***^	0.40^***^	0.72^***^	0.46^***^	0.56^***^	0.42^***^	0.48^***^	0.53^***^	**0.62**

^*^p < 0.05.

** *p < 0.01*.

*** *p < 0.001*.

### Verification of the measurement model

**Figure 1:**
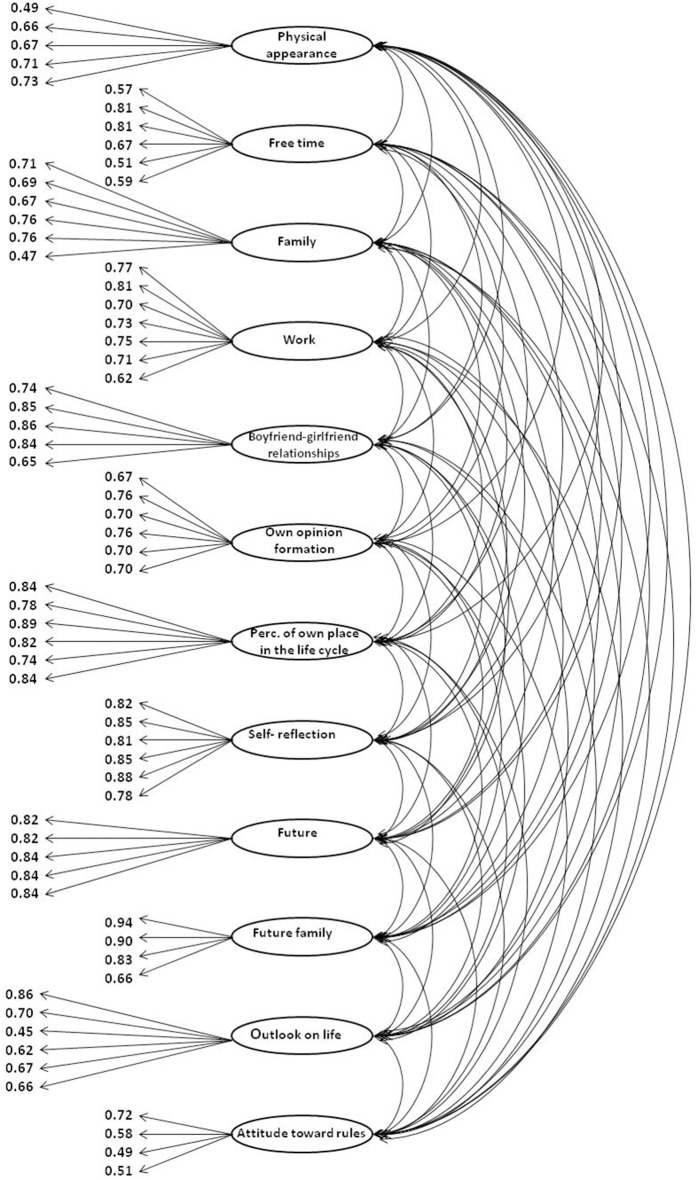
**Confirmatory factor analysis of the Early Identity Exploration Scale**.

We obtained the following model fit indicators: CFI = 0.933, RMSEA = 0.042, 90% CI [0.040, 0.045], χ^2^ = 3660.2, df = 2013. The measurement model of the EIES with factor loadings for all of the items in each scale is depicted in Figure 1. All of the model fits satisfy standard criteria (RMSEA < 0.08 CFI > 0.90) and can thus be accepted.

### Measurement invariance across gender groups

Measurement invariance across gender groups was examined using MGCFA. We obtained the following global fit measures at the configural level: CFI = 0.928, RMSEA = 0.041, 90% CI [0.038, 0.044], χ^2^ = 5546.0, df = 4026; at the metric level: CFI = 0.928, RMSEA = 0.041, 90% CI [0.038, 0.043], χ^2^ = 5613.0, df = 4080; and at the scalar level: CFI = 0.925, RMSEA = 0.041, 90% CI [0.038, 0.043], χ^2^ = 5856.9, df = 4266. Using the common rules promulgated by Chen (2007) configural, metric, and scalar invariance was supported for both gender groups.

### Domain structure identification

According to CFA, the scales were distinguishable. However, most of them were significantly and substantially intercorrelated (see Table 2). This raises the question whether the scales cluster in any higher order factors.

We addressed this question in the additional analysis of the structure of the exploration domains. We conducted EFA on the 12 scales to verify whether higher order factors could be distinguished. More specific, principal axis factor analysis (PAF) with varimax rotation was conducted to assess the underlying structure, and the analysis revealed that the 12 distinguished exploration domains can be grouped into higher order factors. Based on the eigenvalues (>1), there are two factors. The first factor explained 41.8% of the variance, and the second one, 12.0% of the variance. Table 3 presents the loadings of the scales on the factors obtained in EFA. Please note that loadings below 0.40 were omitted and are not reported in the table.

**Table 3:** **Loadings of EIES scales on the factors obtained in exploratory factor analysis**.

	**Factor**	
	**1**	**2**
Work	0.71	
Self-reflection	0.71	
Future	0.67	
Free time	0.66	
Outlook on life	0.61	
Future family	0.59	
Physical appearance	0.53	
Family	0.47	
Own opinion formation		0.74
Perception of own place in the life cycle		0.67
Attitude toward rules		0.58
Boyfriend-girlfriend relationships		0.42

*Loadings below 0.40 were omitted*.

## Discussion

As much research indicates (inter alia: Keyes and Waterman, 2003; Karaś et al., 2015), achieved identity is an important predictor of well-being in adolescence and early adulthood. Therefore, it is worth studying how achieved identity begins to develop in earlier periods of life. Exploration itself does not necessarily predict high well-being. On the contrary, the intensification of identity exploration may even entail a temporary reduction of well-being. However, exploration is an important component of achieved identity; specifically, the step after exploring is commitment making, and achieved identity (developed on the basis of exploration and commitment) leads to well-being. Thus, studying identity exploration—as the first stage leading to achieved identity—is crucial for research on well-being. One of the possible applications of our scale lies in investigating the links between identity and well-being during early adolescence. Additionally, studying exploration in this particular period of life also represents an opportunity to complement and improve identity models in the extant literature. As mentioned above, the 3-dimensional model developed by Crocetti et al. (2008a) tends to focus on commitment and does not investigate the exploration process in a manner that is sufficiently satisfactory and comprehensive for research concerning early adolescence. Another model developing Marcia's conceptualization of exploration and commitment as the primary identity formation processes, the 5-dimensional model presented by Luyckx et al. (2006), distinguishes exploration in breadth from other exploration dimensions but only concentrates on exploration in the area of plans for the future, which can be insufficient in the case of early adolescents. Berzonsky's model (1990) assumes specific styles of exploration in general without dividing this exploration into individual areas or domains. Our proposal fills this gap.

All of the results of the analyses conducted in this study demonstrate the strong psychometric properties of the EIES as a good measurement instrument, and the identity exploration scales achieved high Cronbach's alpha coefficients. The CFA results provide clear support for the theoretical twelve-factor structure of the measurement instrument.

Additionally, we ran EFA on the 12 scales to reveal the domain structure and exploratorily verify whether they cluster into certain sets. We identified two higher order factors: factor 1 comprising eight exploration domains (physical appearance, free time, family, work, self-reflection, future, future family, and outlook on life) and factor 2 consisting of four domains (own opinion formation, perception of own place in the life cycle, attitude toward rules, and boyfriend-girlfriend relationships). All of the involved exploration domains are personal and regard to one's own choices; however, such a division can be interpreted on the basis of a person's individuality and distinctiveness from others. The domains grouped into factor 1 can be described as socializing, whereas for the domains grouped into factor 2, exploration entails even more autonomous and individual decisions than those grouped into factor 1, and they can be described as contesting, changing, and rebellious. Therefore, factor 1 corresponds to metatraits described in personality psychology and denoted by alpha (Digman, 1997) or stability (DeYoung, 2006). Factor 2 corresponds to the metatrait beta (Digman, 1997), which is also referred to as plasticity (DeYoung, 2006). Metatraits are occasionally treated as a frame of reference that allows for the synthesis of knowledge on various phenomena in the field of personality psychology (Strus et al., 2014). Personality features of early exploration and structures of exploration domains are worth studying in future research.

Nevertheless, the fact that CFA revealed satisfactory results and that correlations between the domains did not indicate any redundancies suggests that the 12 domains are still distinguishable at a more narrow level. Therefore, we assume that they can be measured and interpreted separately and not only as a function of two single factors that describe the overall process of exploration in breadth. The measurement invariance that was observed across gender groups further supports the quality of the measurement tool. The study demonstrated that the EIES operates identically for boys and girls.

The primary aim of the study was to expand existing knowledge concerning identity. These results can thus facilitate and enhance further investigations of identity exploration in breadth during early adolescence and encourage further discussion concerning the catalog of exploration domains.

### Conflict of interest statement

The authors declare that the research was conducted in the absence of any commercial or financial relationships that could be construed as a potential conflict of interest.
