# Supplementary material for: The Early Identity Exploration Scale—a measure of initial exploration in breadth during early adolescence
Source: Front Psychol. 2015 Apr 30;6:533. doi: 10.3389/fpsyg.2015.00533 (PMC4415320; doi:10.3389/fpsyg.2015.00533)
Supplement: Supplementary file 1 [file DataSheet1.DOCX]

Appendix 1.

**EIES –version for boys**

English adaptation of polish original questionnaire **(Kłym, Cieciuch, 2014)**

**There are some descriptions of various people below – descriptions of what they do, think or how they feel. You can find statements addressed to you under the descriptions. Please read them carefully, and next to each sentence in the table, tick one answer that describes you best.**

**There are no incorrect answers here – every person is different. We would just like to get to know you, so please tell us about yourself frankly.**

**1.**

| Simon doesn’t pay a lot of attention to what he wears every day. How to dress is not important to him. He doesn’t consider what kind of style could be suitable for him or experiment with his image and appearance.  Jack recently started to dress differently than before. Issues like outfit or hairstyle have become important to him. He tries to find his own style and talks to others about his look. |
| --- |

*Now, please describe yourself. Do you often think about your appearance?*

*Answer each sentence by marking an X in the appropriate column of the table below.*

|  | very rarely or never | rarely | sometimes | often | very often or always |
| --- | --- | --- | --- | --- | --- |
| 1. I recently began to dress differently than before. |  |  |  |  |  |
| 1. I would like to find my own clothing style. |  |  |  |  |  |
| 1. I pay a lot of attention to my hairstyle. |  |  |  |  |  |
| 1. I talk to others about my look. |  |  |  |  |  |
| 1. In magazines and on the Internet, I look for ideas of various outfits and hairstyles to choose the ones that are most suitable for me. |  |  |  |  |  |

| Jim would like to participate in some extra classes to get to know what he is good at. In his free time, he does a lot of things to find out what activities are interesting to him and which are not. Discovering his passions is important to him.  Steve doesn’t think about what activities he is good at. He knows what he likes, and that is what he does in his free time without exploring other ways to spend his free time. |
| --- |

**2.**

*Now, please describe yourself. What do you do in your free time?*

*Answer each sentence by marking an X in the appropriate column of the table below.*

|  | very rarely or never | rarely | sometimes | often | very often or always |
| --- | --- | --- | --- | --- | --- |
| 1. I would like to participate in extra classes to get to know which of them are suitable for me. |  |  |  |  |  |
| 1. I would like to discover some of my passions and interests that I don’t know yet. |  |  |  |  |  |
| 1. I feel the need to discover my strengths. |  |  |  |  |  |
| 1. I look for new forms of fun and entertainment. |  |  |  |  |  |
| 1. I participate in extra classes to check what I’m good at. |  |  |  |  |  |
| 1. I discover new activities that give me joy. |  |  |  |  |  |

**3.**

| Matt has thought about his relatives a lot recently. He has thought about who he would be if he were born in a different family. He wonders whether he matches his family or not and how is he similar to particular family members.  Bart does not wonder how would his life look like if his parents and siblings were different. He does not think about the relationships in his family nor talk to others about that. |
| --- |

*Now, please describe yourself. Do you think a lot about your family?*

|  | very rarely or never | rarely | sometimes | often | very often or always |
| --- | --- | --- | --- | --- | --- |
| 1. I consider the extent to which I match my family. |  |  |  |  |  |
| 1. I wonder who I would be if I were born in a different family. |  |  |  |  |  |
| 1. I compare my family to the families of my friends and colleagues. |  |  |  |  |  |
| 1. I think about how my life would be if my parents were different. |  |  |  |  |  |
| 1. I think about relationships in my family. |  |  |  |  |  |
| 1. I talk to others about my family. |  |  |  |  |  |

*Answer each sentence by marking an X in the appropriate column of the table below.*

**4.**

| Chuck doesn’t think about what he would like to do in his adult life. Right now, the question of what job he will have as an adult is far too distant for him. In his opinion, there will be a proper time for that.  Paul recently began to wonder what he would like to do in the future. He tries to imagine himself in various jobs and considers which one would be the most suitable for him. He talks to others about this and searches for information on the Internet and in books. |
| --- |

*Now, please describe yourself. Do you think about your future job?*

*Answer each sentence by marking an X in the appropriate column of the table below.*

|  | very rarely or never | rarely | sometimes | often | very often or always |
| --- | --- | --- | --- | --- | --- |
| 1. I recently began to wonder what I would like to do in the future. |  |  |  |  |  |
| 1. I consider how I would like to live. |  |  |  |  |  |
| 1. I try to imagine myself in various jobs. |  |  |  |  |  |
| 1. I wonder what kind of job would be the most suitable for me. |  |  |  |  |  |
| 1. I feel that the proper time to think about job I would like to get in the future is right now. |  |  |  |  |  |
| 1. I talk to others about what kind of job would be good for me in the future. |  |  |  |  |  |
| 1. I look on the Internet, in books, etc. for some information on various professions and kinds of future jobs. |  |  |  |  |  |

**5.**

| Recently, Patrick has started thinking more about girls than he did before. He wonders what sort of girl would be the best for him. He also thinks about what kind of relationship he would like to create and talks to others about his relationships with girls.  Alex doesn’t have a girlfriend, and he hasn’t thought about what his perfect girl would be like. He also doesn’t think about what type of boyfriend-girlfriend relationship would be suitable for him. Romantic relationships do not seem relevant to him right now. |
| --- |

*Now, please describe yourself. Do you think about your relationships with girls?*

*Answer each sentence by marking an X in the appropriate column of the table below.*

|  | very rarely or never | rarely | sometimes | often | very often or always |
| --- | --- | --- | --- | --- | --- |
| 1. I think about girls. |  |  |  |  |  |
| I recently started to pay more attention to girls and their looks, outfits and personality. |  |  |  |  |  |
| 1. I wonder what kind of girl would be the best for me. |  |  |  |  |  |
| 1. I think about what type of boyfriend-girlfriend relationship would be the most suitable for me. |  |  |  |  |  |
| 1. I talk to others about my relationships with girls. |  |  |  |  |  |

**6.**

| Lucas, more often than before, notices that his opinion on many issues is different than his parents’ opinion. When he agrees with adults, he admits they are right; when he disagrees, he shows it.  Tom agrees with his parents on many issues and very rarely has different opinions. He doesn’t oppose his parents and rarely considers whether his parents could be wrong. |
| --- |

*Now, please describe yourself. Do you often have a different opinion than adults do?*

*Answer each sentence by marking an X in the appropriate column of the table below.*

|  | very rarely or never | rarely | sometimes | often | very often or always |
| --- | --- | --- | --- | --- | --- |
| 1. Before I follow my parents’ directions, I consider whether they make sense. |  |  |  |  |  |
| 1. I wonder if my parents are always right. |  |  |  |  |  |
| 1. I have different opinions from my parents. |  |  |  |  |  |
| 1. I try to convince my parents of my opinion. |  |  |  |  |  |
| 1. If I have different opinion than adults do, I show it. |  |  |  |  |  |
| 1. I feel opposition to what my parents say. |  |  |  |  |  |

**7.**

| Martin doesn’t mind when his parents or other adults treat him like a child. He understands he isn’t grown-up yet, and because of that adults shouldn’t deal with him like an equal.  Tony doesn’t like when parents treat him as if he were a child because he no longer feels like one. Some things he used to like now seem too childish. He hasn’t felt like a child for some time, and he doesn’t like it when adults treat him like a child. |
| --- |

*Now, please describe yourself. Do you feel like a child ?*

*Answer each sentence by marking an X in the appropriate column of the table below.*

|  | very rarely or never | rarely | sometimes | often | very often or always |
| --- | --- | --- | --- | --- | --- |
| 1. It makes me upset when somebody treats me like a child. |  |  |  |  |  |
| 1. I feel I’m no longer a child. |  |  |  |  |  |
| 1. I mind being treated like a child. |  |  |  |  |  |
| 1. I think I’m mature and in many cases I can decide for myself. |  |  |  |  |  |
| 1. I feel I know what is good for me better than adults do. |  |  |  |  |  |
| 1. I think adults should treat me more seriously. |  |  |  |  |  |

**8.**

| Billy recently began to think about himself more. He asks himself a lot of questions and cares about knowing himself and getting to know what kind of person he is.  Ed doesn’t spend his time wondering about himself. He knows who he is and doesn’t think about it. He feels no need to find out new things about himself. |
| --- |

*Now, please describe yourself. Do you often think about yourself?*

*Answer each sentence by marking an X in the appropriate column of the table below.*

|  | very rarely or never | rarely | sometimes | often | very often or always |
| --- | --- | --- | --- | --- | --- |
| 1. I ask myself questions about myself. |  |  |  |  |  |
| 1. I wonder who I really am. |  |  |  |  |  |
| 1. I feel the need to find out new things about myself. |  |  |  |  |  |
| 1. I reflect on myself. |  |  |  |  |  |
| 1. I care about having accurate knowledge of myself. |  |  |  |  |  |
| 1. I feel the need to talk to someone about what I am and who I am. |  |  |  |  |  |

**9.**

| Mark often ponders the various directions he could take in his life. He reflects on how he would like to live and which lifestyle would be good for him. He seriously deliberates on his plans for the future.  Adam doesn’t think about things he could do in the future. He claims it’s not worth worrying about important things in advance. He doesn’t consider his future or goals he could set at all. These matters seem too distant to him. |
| --- |

*Now, please describe yourself. Do you think about the future?*

*Answer each sentence by marking an X in the appropriate column of the table below.*

|  | very rarely or never | rarely | sometimes | often | very often or always |
| --- | --- | --- | --- | --- | --- |
| 1. I seriously deliberate on various directions I could take in my life. |  |  |  |  |  |
| 1. I think about various things I could do in the future. |  |  |  |  |  |
| 1. I consider many lifestyles that could suit me. |  |  |  |  |  |
| 1. I think about various goals I could set. |  |  |  |  |  |
| 1. I think about various lifestyles that could be good for me. |  |  |  |  |  |

**10.**

| Philip claims it’s too soon to think about the family he will build someday in the future. He doesn’t reflect on what this family will be like or what kind of relations there will be between the members.  Chris recently started to be interested in what his future family will be like and frequently reflects on it. He thinks and talks to others about how he would like his future family to function. |
| --- |

*Now, please describe yourself. Do you think about your future family?*

*Answer each sentence by marking an X in the appropriate column of the table below.*

|  | very rarely or never | rarely | sometimes | often | very often or always |
| --- | --- | --- | --- | --- | --- |
| 1. I’m interested in what my future family will be like. |  |  |  |  |  |
| 1. I think about what kind of family I will form someday. |  |  |  |  |  |
| 1. I reflect on the relationships I will build in the family. |  |  |  |  |  |
| 1. I talk to others about how I would like my future family to be like. |  |  |  |  |  |

**11.**

| Peter has recently started to doubt what he believes in, and so he reflects a lot about what his values are and talks to others about them. These reflections and conversations allow him to deepen his own beliefs.  Mike believes it’s better to stick to established principles and beliefs than to think of different value systems. He doesn’t feel the need to verify what he believes in, and so he doesn’t look for information that would help him to justify his beliefs and values. |
| --- |

*Now, please describe yourself. Do you deliberate on what you believe in?*

*Answer each sentence by marking an X in the appropriate column of the table below.*

|  | very rarely or never | rarely | sometimes | often | very often or always |
| --- | --- | --- | --- | --- | --- |
| 1. I reflect on which values are important to me. |  |  |  |  |  |
| 1. I have doubts about my beliefs. |  |  |  |  |  |
| 1. I think sometimes it’s good to consider if someone who e.g., believes in another god can be right. |  |  |  |  |  |
| 1. I talk to others about what I believe in. |  |  |  |  |  |
| 1. Conversations with others allow me to deepen and expand my beliefs. |  |  |  |  |  |
| 1. I read and look for information to elaborate my own system of values and beliefs. |  |  |  |  |  |

**12.**

| Bruce thinks that rules and regulations are important and that you should always follow them. In his opinion, rules help people in their lives. He doesn’t consider whether all binding regulations make sense.  Max wonders whether all rules, prohibitions, and orders are needed. Sometimes he breaks the rules if he doesn’t see the point of them. He talks to others about this and questions established rules. |
| --- |

*Now, please describe yourself. Do you consider whether rules are needed?*

*Answer each sentence by marking an X in the appropriate column of the table below.*

|  | very rarely or never | rarely | sometimes | often | very often or always |
| --- | --- | --- | --- | --- | --- |
| 1. I wonder whether some rules and regulations make sense. |  |  |  |  |  |
| 1. I consider what would happen if I didn’t follow the rules. |  |  |  |  |  |
| 1. It happens that I break some rules. |  |  |  |  |  |
| 1. I talk to others about whether rules are needed or not. |  |  |  |  |  |
